# Esophagogastroduodenoscopy Screening Intentions During the COVID-19 Pandemic in Japan: Web-Based Survey

**DOI:** 10.2196/40600

**Published:** 2022-11-11

**Authors:** Takemi Akahane, Yasuhiro Nakanishi, Hitoshi Yoshiji, Manabu Akahane

**Affiliations:** 1 Department of Gastroenterology Nara Medical University Kashihara Japan; 2 Department of Health and Welfare Services National Institute of Public Health Wako Japan

**Keywords:** COVID-19, cancer screening, esophagogastroduodenoscopy, EGD, intention, survey, cancer, Japan, screening, men, women, anxiety, information, infection

## Abstract

**Background:**

The number of people undergoing cancer screening decreased during the COVID-19 pandemic. The pandemic may have affected the willingness and motivation of undergoing cancer screening by those eligible for it.

**Objective:**

This study aims to clarify the effect of the COVID-19 pandemic on the intention to undergo cancer and esophagogastroduodenoscopy (EGD) screening.

**Methods:**

We performed a web-based survey on the intention to undergo screening among 1236 men and women aged 20-79 years. The numbers of participants by sex and 10-year age groups were equal. The survey was conducted in January 2021, during which the government declared a state of emergency because of the third wave of the COVID-19 pandemic in Japan. Emergency declarations were issued in 11 prefectures among all the 47 prefectures in Japan.

**Results:**

In total, 66.1% (817/1236) of the participants felt anxious about undergoing screening due to COVID-19. More women than men were anxious about undergoing screening. By modality, EGD had the highest percentage of participants with anxiety due to COVID-19. Regarding the intention to change the participants’ appointment for screening, the most common strategies were to book an appointment for a time during nonpeak hours, postpone the appointment to a later date, and change the mode of transportation. In addition, 35.8% (442/1236) of the participants were willing to cancel this year’s screening appointment. Among the 1236 participants, 757 (61.2%) were scheduled for screening in 2020. Of the 757 participants in this subgroup, 68% (n=515) did not change the schedule, 6.1% (n=46) cancelled, and 26% (n=197) made some changes, including changing the appointment date, hospital, or mode of transportation. Among the 296 participants scheduled for EGD screening, 18.9% (n=56) made some changes, 5.7% (n=17) cancelled on their own, and 2.7% (n=8) cancelled on the hospital’s order. Based on the previous screening results, the percentage of participants who felt anxious about EGD due to the COVID-19 pandemic was higher in the order of those who had not undergone screening and those who were judged to be in need of further examination in screening but did not visit a hospital for it. In the logistic regression analysis, the factors associated with anxiety about EGD screening due to the COVID-19 pandemic were “viral infection prevention measures,” “waiting time,” “fees (medical expenses),” “mode of transportation,” “worry about my social position if I contracted COVID-19,” and “perceived the risk of gastric cancer.” However, “residence in declared emergency area” was not associated with EGD anxiety due to COVID-19.

**Conclusions:**

Excessive anxiety about COVID-19 may lead to serious outcomes, such as a “decreasing intention to undergo EGD screening,” and it is necessary to thoroughly implement infection prevention measures and provide correct information to examinees.

## Introduction

The COVID-19 pandemic has led to severe restrictions in almost all countries and has affected many health care services worldwide. It disrupted the use of preventive health care services. In the United States, the American College of Radiology supported the postponement and rescheduling of nonurgent care, including cancer screening [1]. Screening for cancer is a proven and recommended approach to prevent deaths owing to cancer. The number of people undergoing cancer screening decreased during the COVID-19 pandemic [2]. Although there was an increase in the number of cancer screening tests beginning in late 2020, screenings remained between 29% and 36%, lower than those in the prepandemic era [3]. Coma et al [4] reported that during the pandemic, the number of malignant neoplasms decreased in all age groups, and the number of colonoscopies and mammograms also decreased. However, the number of chest radiographies increased. Another study conducted in north-eastern United States during the COVID-19 pandemic revealed a significant decrease in the number of patients undergoing screening tests for cancer and in the number of ensuing diagnoses of cancerous and precancerous lesions [5]. According to a survey conducted by the Japan Cancer Society, the number of people undergoing cancer screening in 2020 decreased by 30.5% compared with the number of screenings in the previous year. Consequently, the COVID-19 pandemic could disrupt oncology care by delaying the diagnosis and surgical treatment of cancer owing to reduced screening, thereby leading to the long-term consequence of projected increases in cancer-related deaths [6]. The reduction in the number of cancer screenings has been attributed to health care providers. Health care provider constraints included restrictions on elective procedures and shortages of health care staff owing to redeployment to help with pandemic-related care [7]. At the start of the pandemic, elective medical procedures, including cancer screening, were put on hold to conserve medical resources and reduce the risk of spreading COVID-19 in health care settings. However, health systems are now back to scheduling cancer screening tests and examinations. Even when health care providers have increased the availability of preventive care and cancer screenings, many patients face constraints such as loss of income and employer-based insurance coverage [2] and fear of contracting COVID-19 during in-person health care visits [8]. To increase the number of people who receive screening while the COVID-19 pandemic continues, it is necessary to survey the intention to be screened. However, to our knowledge, no studies have investigated the causes of refraining from undergoing cancer screening because of the effect of the COVID-19 pandemic.

This study aimed to examine the predictors of anxiety around cancer screening owing to the COVID-19 pandemic, with a focus on esophagogastroduodenoscopy (EGD).

## Methods

### Survey Method and Participants

All participants were recruited using an internet panel survey company, as we have previously reported [9-11]. All participants were registered as panel members with the company. The participants of this study included registered panel members aged between 20 and 79 years. First, to recruit participants, the survey company created a list using random sampling across all registers. Next, an email that gauges interest in survey participation was sent to all the individuals on this list. Registration was ended when the number of participants in each group reached the target sample size to ensure that the number of participants by sex and 10-year age groups was similar. Participants completed and provided their responses via mail. After completing the survey, participants received a small cash reward. This study comprised 1236 participants aged 20-79 years. Each group was balanced for age and sex. Assuming a confidence level of 95%, a margin of error of 5%, and an expected response rate of 50%, the required sample size was calculated to be 384. When the margin of error was assumed to be 3%, the required sample size was calculated to be 1067. Therefore, the sample size of 1236 was considered sufficient for the analysis. The survey was conducted in January 2021, when the Japanese government declared a state of emergency during the third COVID-19 pandemic. Emergency declarations were issued in the following 11 prefectures among all the 47 prefectures in Japan: Tochigi, Saitama, Chiba, Tokyo, Kanagawa, Gifu, Aichi, Kyoto, Osaka, Hyogo, and Fukuoka.

### Survey of Intention to Undergo Screening During the COVID-19 Pandemic

We conducted an internet survey to assess selected measures of interest, that is, sex, age, place of residence, plans to undergo screening or EGD screening in 2020, results of previous screening, anxiety about undergoing screening due to the COVID-19 pandemic, concerns about undergoing EGD screening due to the COVID-19 pandemic, things to be concerned about if you have COVID-19, and whether you feel you are at risk of having gastric cancer (Multimedia Appendix 1).

### Statistical Analyses

Continuous variables were compared between study groups using the *t* test (2-tailed). Categorical variables were compared using a chi-squared test. Logistic regression analysis was performed with anxiety regarding EGD screening due to the COVID-19 pandemic as the dependent variable. The independent variables included anxiety about viral infection control measures, waiting times, fees (medical expenses), mode of transportation, crowdedness, worry about own social position in case of contracting COVID-19, worry about own health in case of contracting COVID-19, worry about family member’s social position in case of contracting COVID-19, worry about health risk to family members in case of contracting COVID-19, perceived risk of contracting gastric cancer, and residence in a declared emergency area.

All statistical analyses were performed using SPSS version 27.0 (IBM Corp). Statistical significance was set at *P*<.05.

### Ethics Approval

This study was approved by the Ethics Committee of the National Institute of Public Health, Japan (NIPH-IBRA#12302, approval date: November 17, 2020). All participants provided informed consent for data collection and storage. Written informed consent for participation in the study was obtained at the time of registration.

### Patient and Public Involvement Statement

Patients or the public were not involved in the design, conduct, reporting, or dissemination plans of our research.

## Results

### Baseline Characteristics of Participants Concerning Anxiety About Screening Due to COVID-19

The background characteristics of the participants are shown in Multimedia Appendix 2. The average age of the participants was 49.4 (SD 16.5) years, with equal numbers in each 10-year age group and both sexes. Moreover, of the 1236 participants, 63.3% (n=783) resided in a declared emergency area. Furthermore, 66.1% (n=817) responded that they were anxious about undergoing screening due to the COVID-19 pandemic. There were more women than men in the group who were anxious about undergoing screening, but there were no significant differences in age or the percentage of people who resided in a declared emergency area (Table 1).

**Figure 1:**
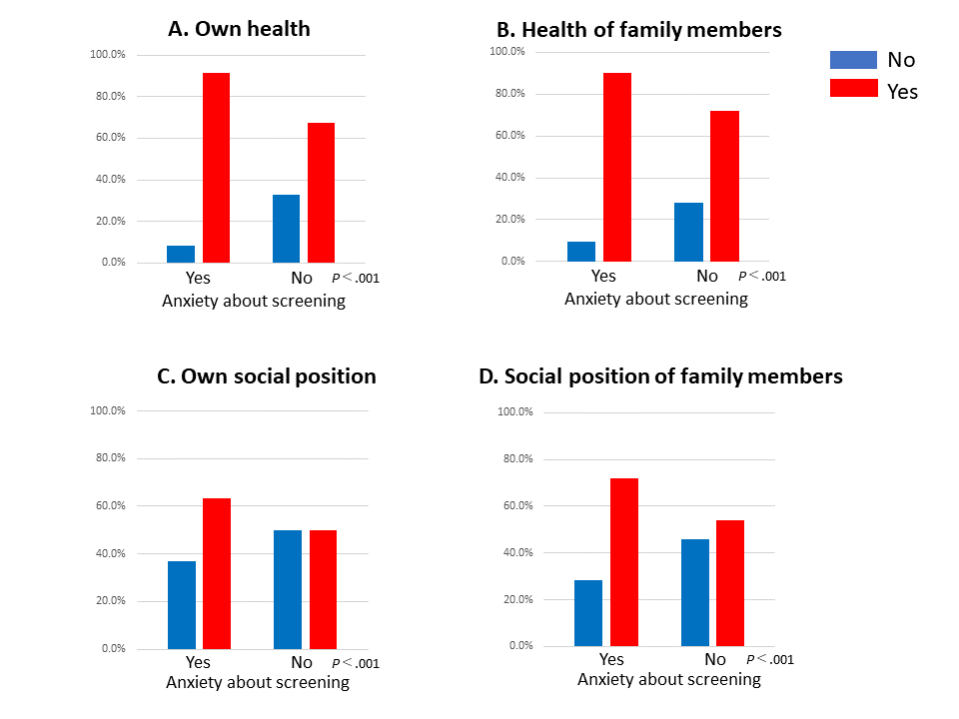
Association between anxiety due to having COVID-19 and intention of screening. A. The question was as follows: If you have COVID-19, are you concerned about your health? B. The question was as follows: If you have COVID-19, are you concerned about the health of your family members? C. The question was as follows: If you have COVID-19, are you concerned about your social status? D. The question was as follows: If you have COVID-19, are you concerned about the social status of your family members?

Participants who were anxious about receiving screening due to COVID-19 were significantly more likely to worry about their own health, the health risk of their family members, their own social position, or the social position of their family members if they had COVID-19 compared with those who were not anxious (Figure 1).

Excessive crowdedness was the most common concern regarding screening (n=1036, 83.8%), followed by waiting time (n=966, 78.2%), viral infection control measures (n=958, 77.5%), transportation (n=786, 63.6%), and fees (n=734, 59.4%; Table 2). By modality, the percentage of participants who felt anxious because of COVID-19 was higher for EGD and colonoscopy (Table 3).

Regarding the intention to change the screening, the most common strategies were to book an appointment for a time during nonpeak hours, postpone the appointment to a later date, and change the mode of transportation. In addition, 35.8% (442/1236) of the participants were willing to cancel this year’s checkup (Table 4).

Among the 1236 participants, 757 (61.2%) were scheduled for screening in 2020. In this subgroup of 757 participants, 68% (n=515) did not change the schedule, 6.1% (n=46) cancelled, and 26% (n=197) made some changes, such as booking an appointment for a time during the nonpeak hours, postponing the appointment to a later date, or changing the hospital or mode of transportation (Table 5).

Among participants scheduled for screening, 18.9% (56/296) of those scheduled for EGD and 19.6% (37/189) of those scheduled for colonoscopy screening made some changes. Among participants scheduled for EGD, 5.7% (17/296) cancelled on their own, and 2.7% (8/296) cancelled on the hospital’s order (Table 6).

**Table 1:** Anxiety about receiving a screening due to COVID-19 (N=1236).

Characteristics			Anxiety					*P* value	
			Yes (n=817)		No (n=419)				
**Sex, n (%)**									<.001
	Male	372 (45.5)		246 (58.7)					
	Female	445 (54.5)		173 (41.3)					
Age (years), mean (SD)			49.2 (16.2)		49.8 (17.0)		.56		
Declared emergency area, n (%)			528 （64.6）		255 (60.9)		.21		

**Table 2:** Concerns due to the effect of the COVID-19 pandemic in screening.

Concerns	Yes, n (%)	No, n (%)
Crowdedness	1036 (83.8)	200 (16.2)
Waiting time	966 (78.2)	270 (1.8)
Viral infection control measures	958 (77.5)	278 (22.5)
Transportation	786 (63.6)	450 (36.4)
Fees (medical expenses)	734 (59.4)	502 (40.6)

**Table 3:** Anxiety due to the effect of COVID-19 by modality.

Modality	Yes, n (%)	No, n (%)
EGD^a^	510 (41.3)	726 (58.7)
Colonoscopy	485 (39.2)	751 (60.8)
CT^b^	413 (33.4)	823 (66.6)
MRI^c^	409 (33.1)	827 (66.9)
Ultrasonography	402 (32.5)	834 (67.5)

^a^EGD: esophagogastroduodenoscopy.

^b^CT: computed tomography.

^c^MRI: magnetic resonance imaging.

**Table 4:** Intentions to change screening due to the COVID-19 pandemic.

Variable	Yes, n (%)	No, n (%)
Book an appointment for a time during the nonpeak hours	973 (78.7）	263 (21.3)
Postponement	592 (47.9)	644 (52.1)
Change of transportation	577 (46.7)	659 (53.3)
Change to a nearby hospital	546 (44.2)	690 (55.8)
Cancel this year’s screening	442 (35.8)	794 (64.2)
Change to a large hospital	213 (17.2)	1023 (52.8)

**Table 5:** Changes made regarding screening (n=757; multiple answers).

Variable	Value, n (%)
Nothing changed	515 (68)
Book an appointment for a time during the nonpeak hours	95 (12.5)
Postponed	63 (8.3)
Changed to a nearby hospital	45 (5.9)
Changed the mode of transportation	32 (4.2)
Changed to a large hospital	9 (1.2)
Others	7 (1)
Cancelled this year’s screening	46 (6.1)

**Table 6:** Changes made regarding examinations for screening.

Modality	Total participants scheduled for testing, n	Postponed at own will, n (%)	Postponed on hospital order, n (%)	Cancelled at your own will, n (%)	Cancelled on the hospital’s orders, n (%)	No change
EGD^a^	296	20 (6.8)	11 (3.7)	17 (5.7)	8 (2.7)	240 (81.1)
Colonoscopy	189	9 (4.8)	10 (5.3)	14 (7.4)	4 (2.1)	152 (80.4)
CT^b^	216	14 (6.5)	12 (5.6)	8 (3.7)	3 (1.4)	179 (82.9)
MRI^c^	205	8 (3.9)	11 (5.4)	10 (4.9)	6 (2.9)	170 (82.9)
Ultrasonography	341	18 (5.3)	10 (2.9)	17 (5.0)	8 (2.3)	288 (84.5)

^a^EGD: esophagogastroduodenoscopy.

^b^CT: computed tomography.

^c^MRI: magnetic resonance imaging.

### Percentage of Anxiety Stratified by Previous Screening Result

**Figure 2:**
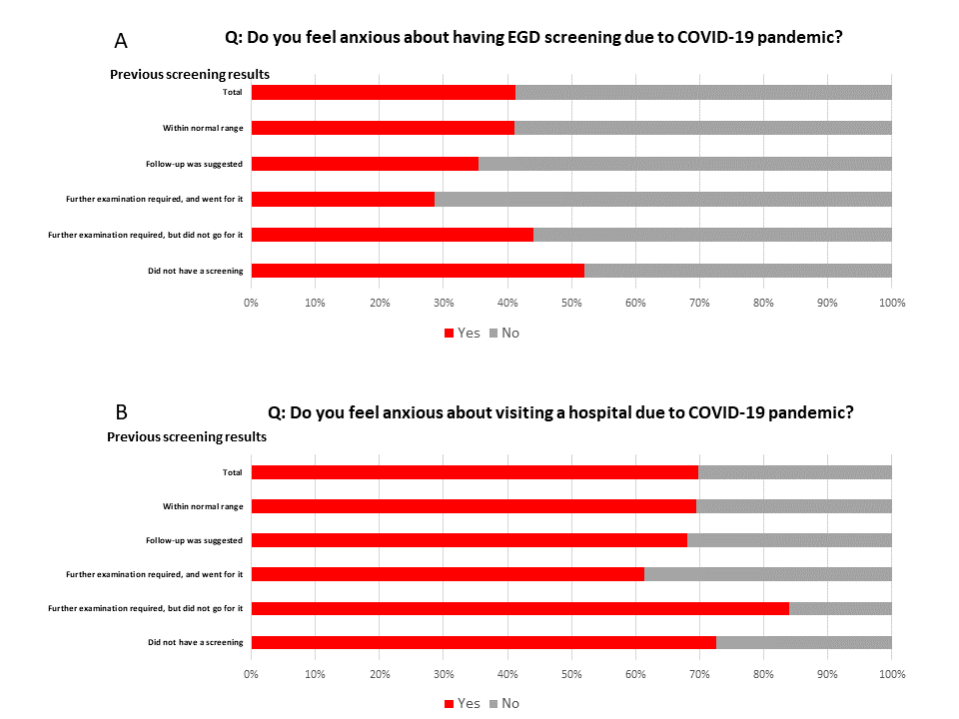
Percentages of respondents reporting anxiety stratified by previous screening result. A. The question was as follows: Do you feel anxious about having EGD screening due to the COVID-19 pandemic? B. The question was as follows: Do you feel anxious about visiting a hospital due to the COVID-19 pandemic? EGD: esophagogastroduodenoscopy.

The proportion of “anxiety about EGD due to the COVID-19 pandemic” responses was analyzed according to the results of the previous screening. Based on previous screening results, participants who had not undergone prior screening had the highest amount of anxiety about EGD screening due to the COVID-19 pandemic (52%). Participants who were judged as needing extended examination but did not go for it had the second highest rate of anxiety about EGD (44%) (Figure 2, section A). Participants who were judged as needing extended examination but did not go for further screening had the highest amount of anxiety about visiting the hospital due to the COVID-19 pandemic (84%). Participants who had not undergone prior screening had the second highest rate of anxiety about visiting the hospital (73%) (Figure 2, section B).

### Feeling at Risk of Developing Gastric Cancer and Anxiety About EGD Screening Due to the COVID-19 Pandemic

**Figure 3:**
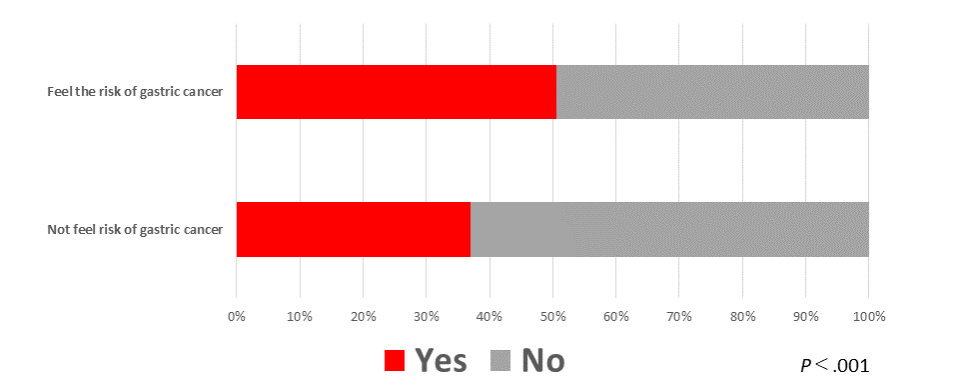
Percentages of respondents with “anxiety about EGD screening” in the “feel the risk of contracting gastric cancer” and the “do not feel the risk of contracting gastric cancer” groups. EGD: esophagogastroduodenoscopy.

We compared “anxiety about EGD screening due to the COVID-19 pandemic’”between participant subgroups classified based on whether or not they felt at risk of contracting gastric cancer. There were 385 participants who felt that they were at risk of contracting gastric cancer, of whom 195 (50.6%) were anxious about EGD screening due to the COVID-19 pandemic. There were 851 patients who did not feel at risk for gastric cancer, of whom 315 (37.0%) were anxious about EGD due to the COVID-19 pandemic. The percentage of “anxiety about EGD screening” was significantly higher in the “feel the risk of contracting gastric cancer” group compared to the “do not feel the risk of contracting gastric cancer” group (Figure 3).

### Factors Associated With Anxiety About EGD Screening Due to the COVID-19 Pandemic

The factors associated with anxiety concerning EGD screening due to COVID-19 were examined using logistic regression analysis (Table 7).

The following factors were related to anxiety regarding EGD screening anxiety due to the COVID-19 pandemic: “viral infection prevention measures,” “waiting time,” “fees (medical expenses),” “mode of transportation,” “worry about my social position if I contracted COVID-19,” and “perceived the risk of gastric cancer.” However, the following responses were not associated with anxiety about EGD due to the COVID-19 pandemic: “residence in declared emergency area,” “worry about my health if I contracted COVID-19,” “crowdedness,” “worry about health risk of my family members if I contracted COVID-19,” or “worry about social position of my family members if I contracted COVID-19.”

**Table 7:** Factors associated with anxiety about esophagogastroduodenoscopy screening due to the COVID-19 pandemic.

Variables	Values	
	Odds ratio (95% CI)	*P* value
Viral infection control measures	3.7 (2.2-6.3)	<.001
Waiting time	2.7 (1.6-4.6)	<.001
Fees (medical expenses)	1.8 (1.4-2.4)	<.001
Mode of transportation	1.7 (1.2-2.3)	.002
Worry about my social position if I contracted COVID-19	1.5 (1.1-2.0)	.008
Perceived the risk of gastric cancer	1.4 (1.1-1.8)	.01
Residence in a declared emergency area	1.2 (0.9-1.6)	.19
Worry about my health if I contracted COVID-19	1.1 (0.73-1.8)	.54
Crowdedness	0.92 (0.51-1.7)	.79
Worry about the health risk of my family members if I contracted COVID-19	0.99 (0.61-1.6)	.98
Worry about the social position of my family members if I contracted COVID-19	1.2 (0.84-1.7)	.32

## Discussion

### Principal Findings

In this study, we conducted a web-based survey on the intention to undergo cancer and EGD screening. In total, 66.1% of participants responded that they felt anxious about undergoing screening owing to the pandemic. With respect to modality, the percentage of participants who felt anxious about screening was the highest for EGD. Factors associated with anxiety around EGD owing to the COVID-19 pandemic were “viral infection prevention measures,” “waiting time,” “fees (medical expenses),” “mode of transportation,” “worry about my social position if I contracted COVID-19,” and “perceived the risk of gastric cancer.” However, residing in a declared emergency area was not associated with anxiety around EGD screening owing to the COVID-19 pandemic. According to a previous screening result, the percentage of “concerned about EGD due to the COVID-19 pandemic” was higher in the groups who had not undergone screening or who needed extended examination but did not undergo it.

The World Health Organization declared the COVID-19 pandemic on March 11, 2020. Plans were put in place to reserve capacity for the surge in COVID-19 clinical care, including the suspension of elective care. In Japan, the Ministry of Health, Labour and Welfare issued a notification, stating that in areas where a state of emergency has been declared, only mass screenings should be postponed during the period the emergency declaration is in effect, and that those who are unable to receive screenings due to postponement will be given another opportunity to receive screening. Hospitals and clinics reduced appointments for cancer screening and nonemergency care to prepare for the diagnosis and treatment of patients with COVID-19 and to prevent the spread of the infection during the periods of emergency declaration, that is, from April to May 2020, and again from January to March 2021. The Japan Cancer Society reported that the number of people receiving cancer screenings in 2020 decreased by 30.5% compared to 2019, and that the number of cancer diagnoses in 2020 was 9.2% lower compared to the previous year (2019). This suggests that the decrease in the number of cancer diagnoses can be attributed to the temporary suspension of cancer screening due to the COVID-19 pandemic and the decrease in the number of people receiving screening due to refraining from visiting hospitals and going outside. In Taiwan, the number of mammography screening examinations decreased in 2020, although the medical system was not disrupted due to the COVID-19 pandemic, likely due to the influence of the population’s perceived risk on their willingness to attend screening [12]. In our survey, 66.1% of the participants felt anxious about undergoing cancer screening regardless of whether they resided in a prefecture where a state of emergency was declared. With the spread of COVID-19, the deterioration of public mental health has become a major global and social problem. A web survey conducted in August 2020 among Japanese participants revealed that 73.2% of the respondents experienced perceived stress related to the COVID-19 pandemic, 34.9% felt intense stress associated with COVID-19, 17.1% were depressed, and 13.5% had severe anxiety symptoms [13]. Therefore, the psychological burden caused by COVID-19 could have affected the intention to undergo screening.

Various factors such as sex, age, marital status, education, occupation and income, place of residence, contact history with patients with COVID-19, and comorbidities were associated with mental health problems such as stress, depression, and anxiety [14-16]. During the COVID-19 pandemic, psychiatric disorders such as depression and anxiety were more prevalent in women than in men [13,17,18]. In this study, more women than men were anxious about undergoing screening. Epidemiological sex differences in anxiety disorders and major depression are well characterized. Anxiety and major depressive disorders are more common in women than in men [19,20]. Besides psychological and cultural factors, biological factors contribute to these sex differences [21]. Therefore, it is likely that there are sex differences in anxiety about undergoing screening owing to COVID-19.

By modality, the percentage of participants who felt anxious due to the COVID-19 pandemic was highest for EGD and colonoscopy, respectively. Malignant neoplasms are the leading cause of death in Japan. Colorectal cancer was the most common cancer type in 2018, followed by gastric cancer. In 2019, colorectal cancer was the second most common cause of cancer-related mortality, followed by gastric cancer. Delays in screening will increase the number of advanced cancers and deaths in the near future.

In a French study investigating the effect of the COVID-19 pandemic on EGD screening in France, 98.7% of endoscopists had cancelled endoscopies, and 73.6% of them had closed the endoscopy outpatient clinic [22]. COVID-19 spreads primarily through droplets of saliva, although airborne transmission and fecal excretion have been documented [23,24]. Severe acute respiratory syndrome coronavirus 2 can survive in the air for several hours [25]. Health care professionals in endoscopy are exposed to COVID-19 through contact with saliva droplets on their face and in airways, via touch contamination, and through contact with a patient’s stool [26,27]. Aerosol infections around endoscopes have also been reported, making EGD among the major aerosol-generating procedures [28,29]. In EGD, where the risk of droplet diffusion and aerosol generation is high, careful measures, such as patient triage and thorough infection protection, are required [30]. Guidelines for endoscopy during the COVID-19 pandemic have been developed [31]. The Japan Gastroenterological Endoscopy Society has published a proposal on its website regarding gastrointestinal endoscopic care for COVID-19. In this survey, we did not ask about the risk for COVID-19 infection from aerosol in EGD, but it is hypothesized that the participants felt anxious about EGD because it is a face-to-face examination compared to other modalities.

In a Japanese study, the Comprehensive Survey of Living Conditions reported a 39% participation rate in gastric cancer screening in 2019. Cancer screening rates in Japan are lower than those in other countries, such as the United Kingdom and Korea. In this study, the percentage of “concerned about EGD due to the COVID-19 pandemic” was higher compared to “haven’t undergone screening” and “needed further examination but did not go for it” based on previous screening results. It is a concern that those who do not undergo screening or visit hospitals for further examination will become increasingly reluctant to do so. In addition, one of the factors associated with EGD screening anxiety due to the COVID-19 pandemic was “perceived to be the risk of gastric cancer.” These results suggest a decrease in the number of gastric cancer screenings and a delay in the detection of gastric cancer. Other factors associated with anxiety around EGD screening due to the COVID-19 pandemic were “viral infection prevention measures,” “waiting time,” “fees (medical expenses),” and “mode of transportation.” Medical institutions and the government must reassure citizens by informing them that appropriate infection prevention measures are being taken during cancer screening.

This study had several limitations. First, we used an internet panel survey company to collect data. While we could obtain responses regarding a wide range of demographic factors such as age, occupation, and income, these groups were not representative of the general population in Japan. However, web surveys have recently become a common method for conducting studies [32,33]. Second, the spread of infection changes daily and varies across regions; however, the survey did not consider this effect. Third, because we did not ask respondents whether they ever had COVID-19, we do not know the effect of the respondents’ personal experiences with previous infection on their anxiety. Finally, the cross-sectional design of this study made it difficult to assess causality.

### Conclusions

This is the first survey-based study to examine the effects of the COVID-19 pandemic on the intention to undergo cancer screening. Most participants were anxious about undergoing screening owing to COVID-19 regardless of whether they resided in a prefecture where a state of emergency was declared, and the percentage of anxiety was higher for EGD than for other modalities. “Viral infection prevention measures,” “waiting time,” “fees (medical expenses),” “mode of transportation,” “worry about my social position if I contracted COVID-19,” and “perceived the risk of gastric cancer” were associated with anxiety about EGD screening anxiety owing to the COVID-19 pandemic. Excessive anxiety about COVID-19 leads to serious outcomes such as delayed detection of cancer and increased cancer-related deaths. Thus, it is necessary to thoroughly implement infection prevention measures and provide correct information to examinees.

## Appendix

Multimedia Appendix 1 Questionnaire survey.

Multimedia Appendix 2 Background characteristics of the participants.

## Abbreviations

EGD: esophagogastroduodenoscopy

## Data Availability

The data sets generated during this study are available from the corresponding author upon reasonable request.
