# Supplementary material for: Esophagogastroduodenoscopy Screening Intentions During the COVID-19 Pandemic in Japan: Web-Based Survey
Source: JMIR Cancer. 2022 Nov 11;8(4):e40600. doi: 10.2196/40600 (PMC9662288; doi:10.2196/40600)
Supplement: Multimedia Appendix 1 [file cancer_v8i4e40600_app1.docx]

Questionnaire Survey

1. Are you anxious about receiving a screening due to the COVID-19 pandemic?
2. Due to the COVID-19 pandemic, are you concerned about the following cause when you go for screening?
3. Waiting time
4. Mode of transportation
5. Viral infection control measures
6. Fees (medical expenses)
7. Crowdedness (i.e., how crowded the waiting room is)
8. Are you anxious due to COVID-19 pandemic when undergoing the following examinations during screening? (Even if you are not actually receiving screening, please assume you are and answer.)
9. Ultrasonography
10. Esophagogastroduodenoscopy
11. Colonoscopy
12. CT
13. MRI
14. Do you want to make the following changes to your screening due to the COVID-19 pandemic?
15. Change to a medical institution nearby
16. Change to a large hospital
17. Book an appointment for a time during the non-peak hours
18. Postpone the appointment to a later date
19. Change the mode of transportation
20. Cancel this year’s checkup
21. Please select the changes you have made to your screening due to the COVID-19 pandemic. (multiple answers allowed)
22. Changed to a medical institution nearby
23. Changed to a large hospital
24. Booked an appointment for a time during the non-peak hours
25. Postponed the appointment to a later date
26. Changed the mode of transportation
27. Other (please specify)
28. Cancelled this year’s checkup
29. Not made any changes
30. No screening scheduled this year
31. Do you feel that you are at risk of contracting gastric cancer?
32. Which one of the below options is applicable with respect to a change in the appointment for the following examinations related to your own screening, due to the COVID-19 pandemic?
     (Options: 1. No change　2. Postponed of my own will　3. Postponed on the hospital’s instruction　4. Cancelled of my own will　5. Cancelled on the hospital’s instruction　6. No such appointment scheduled)
33. Ultrasonography
34. Esophagogastroduodenoscopy
35. Colonoscopy
36. CT
37. MRI
38. If you contracted COVID-19, would you worry a lot about the following matters?
39. Your health
40. Your social position
41. Health risks to family members
42. Family members’ social position
43. Which of the following is relevant to your previous screening result? (choose one)
44. Within normal range.
45. A follow-up was suggested.
46. A further examination was required, and I went for it.
47. A further examination was required, but I did not go for it.
48. I did not have a screening.
