# Supplementary material for: Esophagogastroduodenoscopy Screening Intentions During the COVID-19 Pandemic in Japan: Web-Based Survey
Source: JMIR Cancer. 2022 Nov 11;8(4):e40600. doi: 10.2196/40600 (PMC9662288; doi:10.2196/40600)
Supplement: Multimedia Appendix 2 [file cancer_v8i4e40600_app2.pptx]

## Slide 1
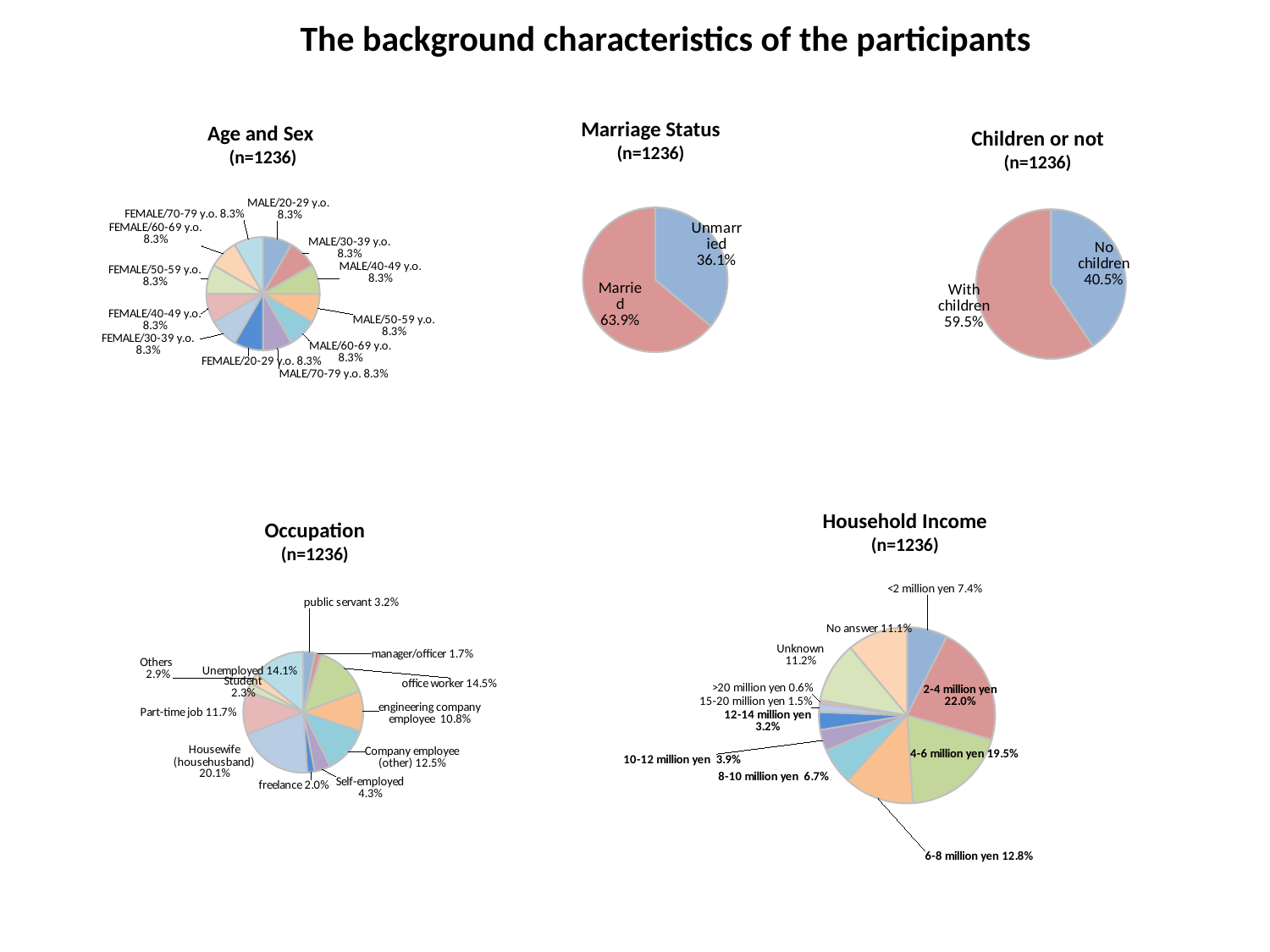

The background characteristics of the participants
Marriage Status
(n=1236)
Age and Sex
(n=1236)
Children or not
(n=1236)
### Chart
| Category | |
|---|---|
| 子供なし | 40.5339805825243 |
| 子供あり | 59.4660194174757 |
### Chart
| Category | |
|---|---|
| 男性/20-29才/全国 | 8.33333333333333 |
| 男性/30-39才/全国 | 8.33333333333333 |
| 男性/40-49才/全国 | 8.33333333333333 |
| 男性/50-59才/全国 | 8.33333333333333 |
| 男性/60-69才/全国 | 8.33333333333333 |
| 男性/70-79才/全国 | 8.33333333333333 |
| 女性/20-29才/全国 | 8.33333333333333 |
| 女性/30-39才/全国 | 8.33333333333333 |
| 女性/40-49才/全国 | 8.33333333333333 |
| 女性/50-59才/全国 | 8.33333333333333 |
| 女性/60-69才/全国 | 8.33333333333333 |
| 女性/70-79才/全国 | 8.33333333333333 |
### Chart
| Category | |
|---|---|
| 未婚 | 36.084142394822 |
| 既婚 | 63.915857605178 |
### Chart
| Category | |
|---|---|
| 200万未満 | 7.44336569579288 |
| 200～400万未満 | 22.0064724919094 |
| 400～600万未満 | 19.4983818770227 |
| 600～800万未満 | 12.7831715210356 |
| 800～1000万未満 | 6.71521035598705 |
| 1000～1200万未満 | 3.88349514563107 |
| 1200～1500万未満 | 3.23624595469256 |
| 1500～2000万未満 | 1.53721682847896 |
| 2000万円以上 | 0.647249190938511 |
| わからない | 11.1650485436893 |
| 無回答 | 11.084142394822 |Household Income
(n=1236)
Occupation
(n=1236)
### Chart
| Category | |
|---|---|
| 公務員 | 3.15533980582524 |
| 経営者・役員 | 1.69902912621359 |
| 会社員(事務系) | 14.4822006472492 |
| 会社員(技術系) | 10.7605177993528 |
| 会社員(その他) | 12.5404530744337 |
| 自営業 | 4.28802588996764 |
| 自由業 | 2.02265372168285 |
| 専業主婦(主夫) | 20.1456310679612 |
| パート・アルバイト | 11.6504854368932 |
| 学生 | 2.26537216828479 |
| その他 | 2.9126213592233 |
| 無職 | 14.0776699029126 |
